# Comparison of sedation between the endoscopy room and operation room during endoscopic submucosal dissection for neoplasms in the upper gastrointestinal tract

**DOI:** 10.1186/s12876-017-0692-8

**Published:** 2017-11-28

**Authors:** Daisuke Yamaguchi, Naoko Yamaguchi, Yuki Takeuchi, Takahiro Yukimoto, Kei Ikeda, Kosuke Matsumoto, Rikako Kinoshita, Saori Kamachi, Kyosuke Sugiyama, Tomohito Morisaki, Keisuke Ario, Hisako Yoshida, Ryo Katsuki, Seiji Tsunada, Kazuma Fujimoto

**Affiliations:** 1 Department of Gastroenterology, National Hospital Organization Ureshino Medical Center, Ureshino, 843-0301 Japan; 2 Department of Anesthesiology, National Hospital Organization Ureshino Medical Center, Ureshino, 843-0301 Japan; 3 Department of Internal Medicine and Gastrointestinal Endoscopy, Saga Medical School, Saga, 849-8501 Japan; 4 Clinical Research Center, Saga Medical School, Saga, 849-8501 Japan

**Keywords:** Propofol, Anesthesia, Desaturation, Perforation, Adverse events

## Abstract

**Background:**

The present study was performed to compare the safety of sedation during endoscopic submucosal dissection (ESD) in the endoscopy room versus operation room.

**Methods:**

In total, 297 patients with gastrointestinal tumors who underwent ESD from January 2011 to December 2016 were retrospectively reviewed. The patients were divided into two groups: those who underwent ESD in the endoscopy room without propofol (Group E) versus operation room with propofol (Group O). The patient, tumor, and procedure characteristics; adverse events; and treatment outcomes were compared between the two groups.

**Results:**

The patient and tumor characteristics, including age (73.6 ± 8.2 vs. 72.5 ± 9.1 years), comorbidities, and tumor size and histology, were not different between Groups E and O. The ESD procedure time was comparable between Groups E and O (105.4 ± 70.4 vs. 106.5 ± 64.4 min), and the anesthesia time was equivalent (138.3 ± 78.1 vs. 148.4 ± 68.8 min). There were no significant differences in adverse events between the two groups. During the ESD procedure, desaturation occurred significantly more often in Group E than O (12.9% vs. 4.0%, *P* = 0.021, odds ratio: 3.53, 95% CI: 1.17–14.4). The recovery time after ESD was significantly longer in Group E than O (180 (100–360) vs. 90 (0–180) min, *P* < 0.001).

**Conclusions:**

A decreased desaturation rate and shorter recovery time after ESD were the advantages of sedation in the operation room with propofol compared with sedation in the endoscopy room. These findings warrant further exploration of the advantages of safe and effective ESD for upper gastrointestinal neoplasms in the operation room.

## Background

Endoscopic submucosal dissection (ESD) is performed to obtain en bloc specimens of early gastric cancer [1–3]. Because ESD involves a longer operation time than conventional endoscopic mucosal resection, multiple doses of anesthetics are required to provide an adequate level of sedation [4]. Sedation methods vary among countries. In Japan, ESD for gastric cancer is usually performed under sedation provided by the endoscopist in the gastrointestinal endoscopy room as described in the Japan Gastroenterological Endoscopy Society guidelines for sedation during gastroenterological endoscopy [5]. ESD usually requires moderate sedation for maintenance of stable sedation levels while avoiding associated complications [6].

The American Society of Anesthesiologists guidelines for sedation by non-anesthesiologists recommend accurate titration of sedation at the level of consciousness for patients undergoing upper endoscopy [7]. In Japan, the endoscopist and/or nursing staff without assistance of the anesthesiologist usually introduces sedation for pain relief in the endoscopy room without expert technical knowledge. In most institutes in Japan, sedation during ESD is performed by intravenous administration of benzodiazepines in combination with opioids [5]. However, this combination often results in over-sedation and/or difficulty maintaining a stable sedation level. The unstable sedation level disturbs the endoscopist, who must concentrate on the ESD procedure [8, 9].

Propofol, a short-acting sedative characterized by rapid recovery, has several advantages including induction of an appropriate sedation level and relative ease of safely maintaining this level. These advantages have led to a worldwide increase in the application of propofol for standard endoscopic procedures [10–14]. Although administration of propofol for ESD, endoscopic retrograde cholangiography, and endoscopic ultrasonography has been described in several reports [6, 8–14], propofol should be administered for sedation only with the permission of the anesthesiologist in Japan.

The aim of the present study was to compare the outcome and complications of gastric ESD under two sedation conditions: i) sedation by benzodiazepines in combination with analgesic agents (opioids) in the endoscopy room; and ii) sedation by propofol in the operation room with the anesthesiologist.

## Methods

### Patients

From January 2011 to December 2016, 297 consecutive patients with early upper gastrointestinal tumors who underwent ESD procedures at the National Hospital Organization Ureshino Medical Center were included in the present retrospective chart review. Patients aged >20 years who fulfilled the following criteria were candidates for the study: i) clinical indications for ESD of early esophageal cancer diagnosed as intramucosal squamous cell carcinoma and ii) diagnosis of early gastric cancer according to the absolute indications and expanded indications in the Gastric Cancer Treatment Guidelines [15]. The exclusion criteria were circumferential lesions of early esophageal cancer and suspected early gastric cancer falling outside of the indications for ESD.

Informed consent for the procedures was obtained from all patients. This retrospective study was conducted according to the Ethical Guidelines for Medical and Health Research Involving Human Subjects. The present study was approved by the Ethics Review Committee of National Hospital Organization Ureshino Medical Center (approval number 16–25).

### Sedation in the endoscopy room

During the observation period, seven endoscopists performed the ESD procedures in either the endoscopy room or operation room based on their judgment. The distribution of the endoscopists was not different between the endoscopy room and the operation room. In the endoscopy room, sedation for the ESD procedures was introduced by the combination of benzodiazepines and opioids. Specifically, diazepam or midazolam was used as a sedative agent, and buprenorphine, pentazocine, or pethidine hydrochloride was used as an analgesic agent. The sedative and analgesic drugs were selected by the individual endoscopist in charge of the patient’s condition.

A dose of 5 to 10 mg of diazepam was infused intravenously [5]. An initial bolus of 3 mg of midazolam for patients with a body weight of <50 kg and 4 mg for patients weighing >50 kg was administered through an intravenous catheter as the main sedative drug, followed by incremental doses (2 mg) when the patient showed signs of discomfort, restlessness, agitation, and/or a response to verbal commands [5]. A midazolam reversal agent was administered after the ESD procedure as necessary. For analgesia, a dose of 0.2 mg of buprenorphine, 15 mg of pentazocine, or 35 mg of pethidine hydrochloride was intravenously injected at the start of ESD. All medications were administered by the physician in the endoscopy division, who did not participate in the actual endoscopic procedure. At least one physician with advanced training in basic and cardiac life support supported the ESD procedure. The anesthesiologist was on standby in case of an emergency but did not support the ESD procedure. Resuscitation devices were equipped in the endoscopy room.

### Sedation in the operation room

In the operation room, the medical agents for sedation during the ESD procedure (propofol for sedation and fentanyl for analgesia) were administered under the anesthesiologist’s supervision. The patient received an initial intravenous bolus of 0.8 mg/kg of 1% propofol emulsion (10 mg/ml). Additional intravenous boluses of 0.5 mg/kg of 1% propofol emulsion were administered until the patient was fully sedated. An automatic infusion pump was applied to maintain a continuous infusion of 3 mg/kg/h and thus maintain the patient’s sedation. The objective was to maintain a sedation level between moderate (proper response to verbal commands either alone or accompanied by light tactile stimulation) and deep (proper response only to repeated or painful stimulation) [6]. As an adjuvant to the sedation, fentanyl was given intravenously at a dose of 1 to 3 μg/kg.

### Monitoring and management of adverse events

In the endoscopy room and operation room, the patient received supplemental oxygen (2 L/min) by a nasal cannula while his or her vital signs and oxygen saturation were continuously monitored and recorded every 5 min using standard three-lead electrocardiography, pulse oximetry, and automatic blood pressure equipment. Chest excursions and the respiratory rate were monitored visually, and the consciousness level was assessed initially after induction of sedation and throughout the ESD procedure. With respect to adverse events, desaturation was defined as a decrease in the oxygen saturation to <90%, hypotension was defined as a decrease in the systolic blood pressure to <80 mmHg, and bradycardia was defined as a decrease in the heart rate to <60 bpm. When oxygen desaturation occurred for >10 s, the supplemental oxygen flow was immediately increased to the >95% saturation level. When the supplemental oxygen did not improve the patient’s oxygenation condition within 3 min, the ESD procedure was interrupted to secure the airway with administration of the reversal agent as necessary. If hypotension developed, the intravenous drip rate was increased by bolus intravenous injection of 8 mg of ephedrine. For treatment of bradycardia, 0.5 mg of atropine sulfate was injected intravenously. In the operation room, these adverse events were managed by the anesthesiologist.

Patients were discharged from the endoscopy room or operation room after the ESD procedure when they showed stable vital signs (blood pressure, oxygen saturation, and heart rate). Vital signs were recorded at the beginning of the ESD procedure until the patient showed sufficient alertness and activity.

### Evaluation of data

The patients were divided into two groups: those who underwent ESD in the endoscopy room (Group E) and those who underwent ESD in the operation room (Group O). The χ^2^ test was used to compare the two groups’ clinical characteristics, including sex, current drinking and smoking habits, American Society of Anesthesiologists classification, and comorbidities (e.g., cardiovascular diseases, cerebrovascular diseases, chronic liver damage, chronic kidney diseases, diabetes mellitus, and hypertension). Continuous variables were summarized as the mean ± standard deviation for a normal distribution, or median [IQR] for a skewed distribution. The patients’ mean age and body mass index were evaluated by Student’s *t*-test. The tumor characteristics, including the location, number, historical classification, macroscopic classification, and depth of invasion, were compared between the two groups by the χ^2^ test. In patients with multiple tumors, the largest tumor was evaluated based on the underlying assumption of statistically independent observations. Anesthetic outcomes (intubation, desaturation, hypotension, bradycardia, and aspiration pneumonia) and treatment outcomes (en bloc resection, delayed hemorrhage, perforation, and mortality) were evaluated by the χ^2^ test. The mean ESD procedure time and anesthesia time were analyzed using Student’s *t*-test. The recovery time was analyzed using the Mann–Whitney U test. The economic outcome including the hospitalization period and cost of hospitalization was analyzed using Student’s *t*-test. Differences were considered statistically significant at *P* < 0.05. Statistical analyses were performed with the statistical analysis software EZR version 1.26 [16].

## Results

Among the 297 patients who underwent ESD procedures, 196 were allocated to Group E and 101 were allocated to Group O. The patients’ baseline characteristics were not significantly different between the two groups (Table 1). The median age of the patients in Groups E and O was 73.6 ± 8.2 and 72.5 ± 9.1 years, respectively. In total, 138 (70.4%) and 78 (77.2%) patients in Groups E and O were male, and 187 (95.4%) and 97 (96.0%) patients in Groups E and O had an American Society of Anesthesiologists physical status of I or II, respectively. Comorbidities included hypertension (58.6%), diabetes mellitus (23.4%), and cardiovascular disease (23.4%).

**Table 1:** Characteristics of patients in the two groups

	Group E	Group O	*P* value
Number of patients (*N*)	196	101	
Age (years)	73.6 ± 8.2	72.5 ± 9.1	0.26
Sex, male	138 (70.4%)	78 (77.2%)	0.22
Drinking	64 (32.6%)	34 (33.7%)	0.90
Smoking	59 (30.1%)	39 (38.6%)	0.15
BMI (kg/m2)	22.2 ± 3.1	22.9 ± 3.1	0.06
ASA classification I-II	187 (95.4%)	97 (96.0%)	1.00
Comorbidity			
Cardiovascular diseases	33 (16.8%)	18 (17.8%)	0.87
Cerebrovascular diseases	23 (11.7%)	12 (11.9%)	1.00
Chronic kidney diseases	6 (3.1%)	8 (7.9%)	0.08
Chronic liver damage	9 (4.6%)	4 (4.0%)	1.00
Diabetes mellitus	40 (20.4%)	29 (28.7%)	0.11
Hypertension	113 (57.6%)	62 (61.4%)	0.62

Group E: patients who underwent endoscopic submucosal dissection in the endoscopy room, Group O: patients who underwent endoscopic submucosal dissection in the operation room, *BMI* body mass index, *ASA* American Society of Anesthesiologists. Values presented as mean ± standard deviation or n (%)

Table 2 lists the characteristics of the gastrointestinal tumors in the two groups. The average tumor size was 17.3 ± 10.5 mm in Group E and 19.7 ± 17.0 mm in Group O, and the ratios of 0-IIc tumors (46.9% vs. 47.5%) and tub1 tumors (58.1% vs. 57.4%) were the highest among all histological subtypes in both groups. There were no significant differences in the baseline tumor characteristics between the two groups.

**Table 2:** Characteristics of tumors in the two groups

	Group E	Group O	*P* value
Size of tumors (mm)	17.3 ± 10.5	19.7 ± 17.0	0.14
Location of tumors			0.10
Esophagus	0 (0%)	2 (2.0%)	
Stomach	196 (100%)	99 (98.0%)	
Upper third	28 (14.3%)	12 (11.9%)	
Middle third	83 (42.3%)	52 (51.5%)	
Lower third	85 (43.4%)	35 (34.7%)	
Number of tumors			0.07
Single	185 (94.4%)	89 (88.1%)	
Multiple	11 (5.6%)	12 (11.9%)	
Histological classification			0.65
0-I	5 (2.6%)	4 (4.0%)	
0-IIa	82 (41.8%)	38 (37.6%)	
0-IIb	5 (2.6%)	6 (5.9%)	
0-IIc	92 (46.9%)	48 (47.5%)	
0-IIa + IIc	11 (5.6%)	5 (5.0%)	
SMT	1 (0.5%)	0 (0%)	
Macroscopic classification			0.25
Adenoma	55 (28.1%)	28 (27.7%)	
Tub1	114 (58.1%)	58 (57.4%)	
Tub2	18 (9.2%)	6 (5.9%)	
por	1 (0.5%)	4 (4.0%)	
Others	8 (4.1%)	5 (5.0%)	
Depth of invasion			0.97
m	174 (88.8%)	91 (90.1%)	
sm	22 (11.2%)	10 (9.9%)	

Group E: patients who underwent endoscopic submucosal dissection in the endoscopy room, Group O: patients who underwent endoscopic submucosal dissection in the operation room. Values are presented as mean ± standard deviation or n (%)

With respect to sedation during the ESD procedures, diazepam (58.2%) or midazolam (44.4%) with buprenorphine (50.5%) was mainly selected for anesthesia in Group E, and propofol with fentanyl was selected for all patients in Group O (Table 3). The anesthesia time was comparable between the two groups (138.3 ± 78.1 vs. 148.4 ± 68.8 min), as indicated in Table 4. The treatment, anesthetic, and economic outcomes of the ESD procedures are shown in Table 4. During ESD, 99.3% of the patients underwent en bloc resection (99.5% in Group E and 99.0% in Group O), and the procedure time was equivalent between Groups E and O (105.4 ± 70.4 vs. 106.5 ± 64.4 min, respectively). There were no significant differences in the outcomes or complications of the ESD procedures between Groups E and O. Specifically, delayed hemorrhage occurred in 5.1 and 1.0% of patients and perforation occurred in 1.5 and 3.0%, respectively. No mortality within 1 month occurred in either group.

**Table 3:** Medications and doses administered during endoscopic submucosal dissection

Anesthesia drug	Group E	Group O
Diazepam	114 (58.2%)	
Mean diazepam dose (mg)	16.1 ± 9.4	
Midazolam	87 (44.4%)	
Mean midazolam dose (mg)	14.1 ± 6.1	
Buprenorphine	99 (50.5%)	
Mean buprenorphine dose (mg)	0.2 ± 0.1	
Pentazocine	52 (26.5%)	
Mean pentazocine dose (mg)	12.7 ± 4.3	
Pethidine hydrochiloride	55 (28.1%)	
Mean pethidine dose (mg)	73.2 ± 24.3	
Propofol		101 (100%)
Mean propofol dose (mg)		633.6 ± 320.0
Fentanyl		101 (100%)
Mean fentanyl dose (μg)		125.0 ± 52.6

Group E: patients who underwent endoscopic submucosal dissection in the endoscopy room, Group O: patients who underwent endoscopic submucosal dissection in the operation room. Values are presented as mean ± standard deviation or n (%)

**Table 4:** Anesthetic and treatment outcomes of endoscopic submucosal dissection

	Group E	Group O	*P* value
Treatment outcome			
Procedure time (min)	105.4 ± 70.4	106.5 ± 64.4	0.89
En bloc resection	195 (99.5%)	100 (99.0%)	1.00
Delayed hemorrhage	10 (5.1%)	1 (1.0%)	0.11
Perforation	3 (1.5%)	3 (3.0%)	0.41
Mortality	0 (0%)	0 (0%)	1.00
Anesthetic outcome			
Anesthesia time (min)	138.3 ± 78.1	148.4 ± 68.8	0.27
Intubation case	0 (0%)	1 (1.0%)	0.34
Desaturation	25 (12.8%)	4 (4.0%)	0.02
Hypotension	26 (13.3%)	8 (7.9%)	0.25
Bradycardia	8 (4.1%)	3 (3.0%)	0.76
Recovery time (min)^a^	180 (100–360)	90 (0–180)	<0.001
Aspiration pneumonia	2 (1.0%)	2 (2.0%)	0.61
Economic outcome			
Hospitalization period (day)	11.3 ± 5.0	10.3 ± 4.6	0.09
Cost of hospitalization (1,000yen)	583.8 ± 273.1	649.4 ± 160.1	0.03

Group E: patients who underwent endoscopic submucosal dissection in the endoscopy room, Group O: patients who underwent endoscopic submucosal dissection in the operation room

^a^Median [interquartile range]; other values are mean ± standard deviation or *n* (%)

In terms of anesthetic outcomes, the anesthesia time was almost identical in the two groups, and most of the patients (99.7%) underwent the ESD procedure without intubation; 0 and 1 patient in Groups E and O required intubation. Desaturation occurred significantly more often in Group E than O (12.8% vs. 4.0%, respectively; *P* = 0.021), whereas there was no significant difference in the occurrence of hypotension or bradycardia between the two groups. The recovery time after the ESD procedure was significantly longer in Group E than O [180 (100–360) vs. 90 (0–180) min, respectively; *P* < 0.001]. There was no significant difference in the occurrence of aspiration pneumonia after the ESD procedures.

In terms of economic outcomes, there were no differences in the hospitalization period for the ESD procedures between Groups E and O (11.3 ± 5.0 vs. 10.3 ± 4.6 days, respectively). The mean cost of hospitalization was significantly lower in Group E than O (583,806.4 ± 273,117.0 vs. 649,415.7 ± 160,137.3 yen, respectively; *P* = 0.027).

## Discussion

In this study, the safety of sedation during ESD in the esophagus and stomach was compared between two sedation conditions: sedation in the endoscopy room and sedation in the operation room. Although ESD has been introduced to obtain en bloc specimens of early-stage gastric cancer in Japan [1, 2, 17–19], ESD requires fine, complicated operative maneuvers with a long period of intraoperative sedation, which might exacerbate the risks of serious complications including perforation and/or bleeding. The present study indicated that sedation with continuous infusion of propofol during ESD in the operation room allows for stable performance of the procedure with a short recovery time. A previous study reported that sedation with propofol for endoscopic procedures leads to a decrease in complications [6, 13, 20]; this was due to the rapid onset and offset of sedation associated with the continuous infusion of propofol.

Sedation for ESD in the operation room might be ideal. ESD procedures in Japan are mainly performed under sedation provided by the endoscopist in the endoscopy room because of space limitations in the operation room and the cost of maintaining a high number of available anesthesiologists. A previous study revealed that administration of propofol by the gastroenterologist did not increase the complication rate [20]. The American Society for Gastrointestinal Endoscopy recommends additional training by the anesthesiologist to ensure safe administration of propofol by non-anesthesiologists during endoscopic procedures [21]. As demonstrated in the present study, sedation with propofol might ideally be administered either by the anesthesiologist or under supervision of the anesthesiologist.

Perforation during ESD is a serious and relatively common complication with an incidence of 3.0 to 4.8% in the stomach [22, 23]. Unstable sedation might result in perforation due to an unexpected gag reflex, hiccups, and/or body movement. In the present study, the en bloc resection rate was 99.3%, the incidence of perforation was 2.0%, and no mortality occurred, and these results were not different between the two sedation groups.

In the present study, desaturation during the ESD procedure occurred significantly more often in the endoscopy room than in the operation room. The increased incidence of desaturation in the endoscopy room might have been due to saliva flowing into the airway, requiring suction of the saliva from the oral cavity during the ESD procedure. The recovery time after ESD was significantly longer in the endoscopic than operation room, although the occurrence of aspiration pneumonia was not different between the two groups. These two factors (desaturation and the recovery time) might be advantages of sedation with propofol in the operation room.

A disadvantage of sedation in the operation room is the high hospitalization fee for ESD in patients with early esophageal or gastric cancers. This high cost is mainly due to the use of general anesthesia, not the duration of hospitalization. Economic evaluation has revealed that ESD performed in the endoscopy room is cost-effective, which might be a problem in terms of health insurance coverage in Japan.

Limitations of this study were the small number of participants, and the fact that it was a single-institution study. In addition, this study was a retrospective nature of the chart review. Consequently, there would have been selection bias of participants by the endoscopists performed the ESD procedures. Further studies involving multiple institutes might be required to accumulate more data on ESD procedures.

## Conclusions

Although decreases in the desaturation rate and recovery time after ESD were the only advantages of sedation with propofol in the operation room, sedation in the operation room might be required to ensure safer application of ESD for tumors of the gastrointestinal tract.

## Abbreviations

ESD: Endoscopic submucosal dissection
Group E: Patients who underwent ESD in the endoscopy room
Group O: Patients who underwent ESD in the operation room
